# DNA methylation-driven gene FAM3D promotes colorectal cancer growth via the ATF4-SESN2-mTORC1 pathway

**DOI:** 10.18632/aging.206115

**Published:** 2024-10-10

**Authors:** Ting Zheng, Ding Zhang, Qingzhen Fu, Mingxue Wang, Zesong Cheng, Yukun Cao, Liwan Wang, Jinyin Liu, Yashuang Zhao

**Affiliations:** 1 Department of Epidemiology, Public Health School of Harbin Medical University, Harbin, Heilongjiang, P.R. China

**Keywords:** colorectal cancer, methylation-driven gene, FAM3D, mTORC1 pathway, prognostic marker

## Abstract

Globally, colorectal cancer (CRC) is the malignant tumor with the highest mortality rate after lung cancer. Abnormal DNA methylation drives dysregulated gene expression, thereby promoting CRC progression and leading to poor prognosis. We identified a 3-CpG methylation signature that is independently associated with CRC prognosis. The model consists of three methylation-driven genes: FAM3 Metabolism Regulating Signaling Molecule D (FAM3D), DAPP1, and PIGR. However, the prognostic significance, biological function, and related mechanisms of the individual methylation-driven gene FAM3D in CRC have not been studied. Here, we discovered that FAM3D expression was reduced in CRC tissues and cells, and that high methylation and low expression of FAM3D were independent prognostic risk factors for CRC. In addition, FAM3D promoted the growth and movement of CRC cells *in vitro* and the proliferation in nude mice, mainly by inhibiting ATF4 transcription and downregulating SESN2 expression, and ultimately activating mTORC1. Furthermore, FAM3D resulted in reduced sensitivity of CRC cells to oxaliplatin, cisplatin, and 5-fluorouracil. Our study showed that FAM3D activates the mTORC1 pathway through the ATF4-SESN2 axis and promotes the malignant progression of CRC, which contributes to predict CRC prognosis and guide individualized treatment.

## INTRODUCTION

Colorectal cancer (CRC) is a major danger to human health, accounting for 10% of cancer incidence and 9.4% of cancer death worldwide [1]. The prognosis for individuals with early-stage CRC is wonderful, but many patients are identified at an advanced stage, with a five-year survival rate of only 14% [2–4]. The pathogenesis of CRC is widely considered to be a multifactorial process involving complex molecular, genetic, and epigenetic changes [5, 6]. Therefore, it is crucial to identify CRC prognostic biomarkers and elucidate their roles and related molecular mechanisms in CRC progression.

DNA methylation is an important epigenetic event that drives tumorigenesis [7, 8]. Methylation-driven genes are those whose expression levels are controlled by DNA methylation. Tumor suppressor genes’ promoter hypermethylation can cause their expression to be silenced, leading to malignant transformation of cell phenotypes and ultimately promoting tumor progression [9, 10]. Many studies have explored the diagnostic value and prognostic significance of methylation-driven genes in solid tumors such as CRC [11, 12]. However, there are few studies on the role of the methylation-driven gene in tumor progression and related molecular mechanisms, which needs to be further explored.

By combining mRNA expression and DNA methylation data, we discovered a 3-CpG methylation predictive model that is independently associated with CRC overall survival (OS) and disease-free survival (DFS). The three methylation-driven genes in the model are *PIGR*, *DAPP1*, and FAM3 metabolic regulator signaling molecule D (*FAM3D*) [13]. These genes play a key role in the occurrence and development of tumors. For example, PIGR can not only promote the malignant progression of hepatocellular carcinoma and pancreatic ductal adenocarcinoma [14–17], but also inhibit cell proliferation and motility in lung cancer, endometrial adenocarcinoma, and CRC [18–20]. Additionally, knocking down *DAPP1* significantly inhibits the growth of *EGFR* mutant lung adenocarcinoma cells [21].

Among the three, *FAM3D* is particularly noteworthy. As a member of the FAM3 gene family, the production and secretion of intestinal-derived protein encoded by *FAM3D* are regulated by nutritional status [22, 23]. FAM3D regulates intestinal inflammation and maintains intestinal homeostasis through FPR1 and FPR2 receptors [24]. A study showed that the deletion of *FAM3D* can promote the progression of colon inflammation-related carcinogenesis in mice [25]. In addition, FAM3D can also inhibit the proliferation and migration of squamous cell carcinoma cells and reduce their resistance to chemotherapy drugs [26, 27].

However, no studies have yet explored the effects of the individual methylation-driven gene *FAM3D* on CRC prognosis, as well as its function and related molecular mechanisms in CRC progression. Considering the important role of FAM3D in maintaining intestinal homeostasis, preventing inflammation-related carcinogenesis, and inhibiting cancer cell proliferation and migration, it is of great significance to study its specific functions and mechanisms in CRC. By revealing the biological functions and related mechanisms of FAM3D in CRC, we can not only further understand the pathogenesis of CRC, but also provide new targets for personalized treatment and prognosis evaluation.

Hence, our study evaluated the methylation, expression, and associated prognostic value of *FAM3D* in CRC patients. Next, we generated CRC cells with *FAM3D* knockout (KO) or overexpression to study the effects of FAM3D on CRC cell functions and related regulatory mechanisms.

## RESULTS

### Methylation, expression, and clinical relevance of *FAM3D* in CRC patients

We have reported that the 3-CpG methylation prognostic model consisting of 3 methylation-driven genes (*FAM3D*, *DAPP1*, and *PIGR*) is an independent prognostic biomarker for CRC [13]. Based on this, our study further explored the prognostic significance of *FAM3D* in CRC as well as its impact on CRC cell functions and potential molecular mechanisms. The relevant workflow is shown in Supplementary Figure 1.

**Figure 1:**
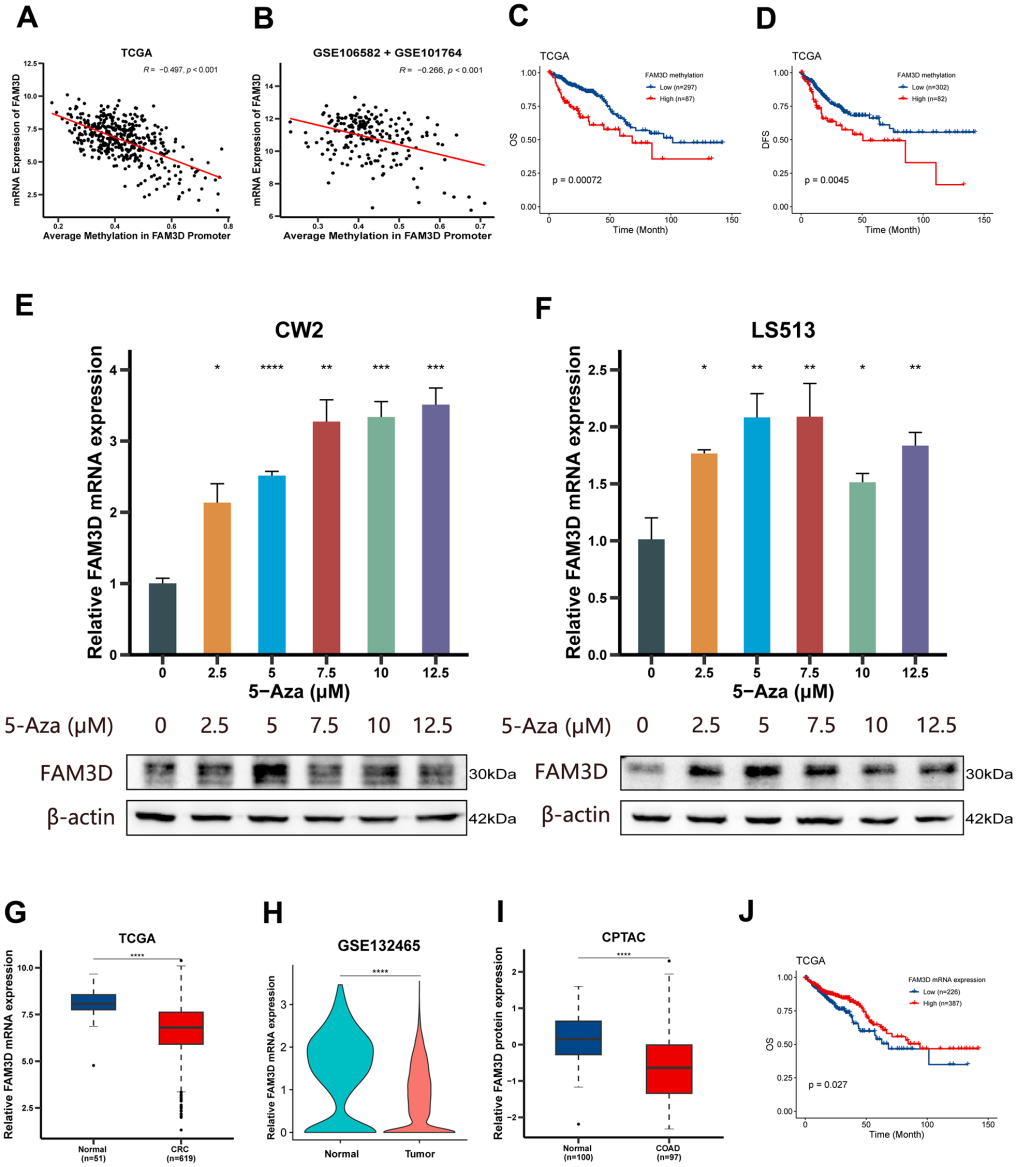
**Methylation, expression and prognosis of *FAM3D*.** (**A**, **B**) Correlations between *FAM3D* mRNA expression and methylation of the *FAM3D* promoter in the (**A**) TCGA and (**B**) GEO databases (GSE106582 + GSE101764). (**C**, **D**) Kaplan-Meier curves of (**C**) OS and (**D**) DFS based on the methylation of the *FAM3D* promoter in tumor tissues from the TCGA cohort. (**E**, **F**) Relative *FAM3D* mRNA and protein expression after treatment with a series of concentrations of 5-Aza in (**E**) CW2 and (**F**) LS513 cells (differences compared to the DMSO group). (**G**) The mRNA expression of *FAM3D* in normal tissues and CRC tissues in the TCGA cohort. (**H**) The mRNA expression of *FAM3D* in normal cells and CRC cells in GSE132465. (**I**) The protein expression of *FAM3D* in normal tissues and COAD tissues in the CPTAC cohort. (**J**) Kaplan-Meier curve of OS based on *FAM3D* mRNA expression in tumor tissues from the TCGA cohort. *P < 0.05; **P < 0.01, ***P < 0.001, ****P < 0.0001.

First, we examined the connection between the mRNA level and *FAM3D* promoter methylation using TCGA and GEO. The findings suggested an inverse relationship between *FAM3D* expression and methylation of its promoter region (rTCGA = -0.497 and rGSE106582+GSE101764 = -0.266; rGSE131013+GSE44076 = -0.405) (Figure 1A, 1B and Supplementary Figure 2A). Prognostic analysis found that hypermethylation of *FAM3D* not only resulted in decreased OS but also contributed to reduced DFS (Figure 1C, 1D). *FAM3D* methylation also acted as an independent predictor of the prognosis of CRC patients (HRTCGA (95% CI) = 2.02 (1.23-3.34), *P* = 0.006) (Supplementary Table 1). Next, we treated CRC cell lines (CW2 and LS513) with a series of concentrations of 5-Aza (Macklin) and examined *FAM3D* expression. Results showed elevated levels of *FAM3D* mRNA and protein after demethylation (Figure 1E, 1F).

We further explored *FAM3D* expression in CRC tissues in TCGA and GEO, which found that *FAM3D* mRNA was significantly reduced in CRC tissues (Figure 1G and Supplementary Figure 2B, 2C). In addition, consistently, *FAM3D* mRNA in CRC cells was significantly downregulated (Figure 1H), and it was mainly expressed in epithelial cells (Supplementary Figure 2D). Furthermore, according to the CPTAC database, FAM3D protein level was significantly reduced in colon cancer (COAD) tissues (Figure 1I). Although the expression of the *FAM3D* mRNA did not obviously change (Supplementary Figure 2E), FAM3D protein was significantly reduced in advanced-stage patients (Supplementary Figure 2F). Subsequent investigations showed that increased *FAM3D* mRNA expression was positively correlated with better OS and DFS (Figure 1J and Table 1 and Supplementary Figure 2G–2J). *FAM3D* mRNA was also discovered to be an independent prognostic factor for CRC patients (Supplementary Tables 2–4).

**Table 1:** Associations between *FAM3D* mRNA and clinicopathological features in CRC patients of TCGA cohort.

**Variables**	**Number (%)**	***FAM3D* mRNA expression**		***P-*Value**
		**Low**	**High**	
**Age**				
Age ≥ 60 years	416 (70.15%)	160	256	1
Age < 60 years	177 (29.85%)	68	109	
**Gender**				
Male	321 (54.13%)	116	205	0.241
Female	272 (45.87%)	112	160	
**T stage**				
T3-4	467 (79.29%)	178	289	0.886
T1-2	122 (20.71%)	48	74	
**N stage**				
N1-3	253 (43.10%)	100	153	0.665
N0	334 (56.90%)	125	209	
**M stage**				
M1	82 (15.86%)	36	46	0.371
M0	435 (84.14%)	165	270	
**Type**				
COAD	436 (73.52%)	173	263	0.352
READ ^a^	157 (26.48%)	55	102	
**OS Status**				
Dead	124 (20.91%)	58	66	**0.041^b^**
Alive	469 (79.09%)	170	299	

^a^READ, rectum cancer; ^b^the bold values mean the difference is statistically significant.

### FAM3D promotes the malignant phenotypes of CRC cells *in vitro*

**Figure 2:**
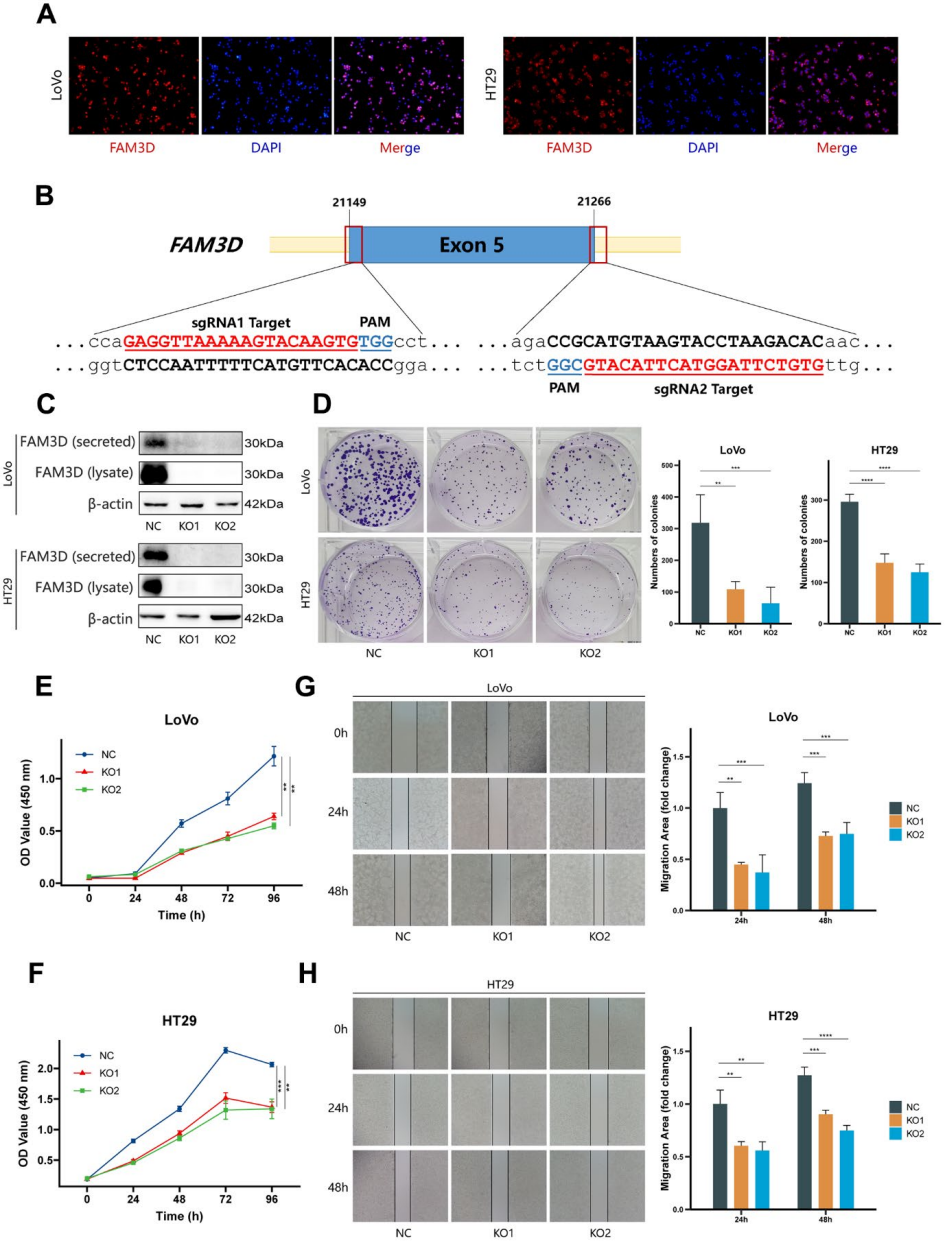
**FAM3D promotes CRC cell proliferation, migration, and invasion *in vitro*.** (**A**) The localization of FAM3D in LoVo and HT29 cells. (**B**) Schematic diagram of sgRNA targeting the human *FAM3D* gene locus. Two sgRNA sequences, sgRNA1 and sgRNA2, are marked in red, and the protospacer adjacent motif (PAM) sequences are presented in blue. (**C**) Western blot analysis of FAM3D in the supernatant (secreted) and cell lysate (lysate) of LoVo and HT29 cells after *FAM3D* KO. (**D**–**F**) Cell proliferation was detected by (**D**) colony formation assay and (**E**, **F**) CCK-8 assay in LoVo and HT29 cells. (**G**, **H**) Cell migration was detected by a wound healing assay in (**G**) LoVo and (**H**) HT29 cells. **P < 0.01, ***P < 0.001, ****P < 0.0001.

**Figure 3:**
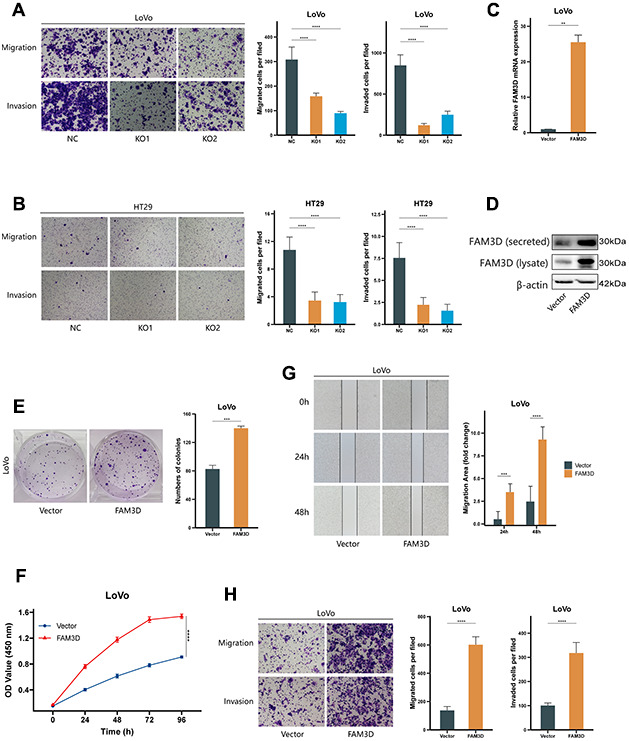
**FAM3D promotes CRC cell proliferation, migration, and invasion *in vitro*.** (**A**, **B**) Migration and invasion were detected by transwell assays in (**A**) LoVo and (**B**) HT29 cells. (**C**, **D**) Overexpression of *FAM3D* was confirmed by (**C**) RT-qPCR and (**D**) Western blot in LoVo cells. (**E**, **F**) Proliferation was detected after *FAM3D* overexpression. (**G**, **H**) Migration and invasion were detected after *FAM3D* overexpression. **P < 0.01, ***P < 0.001, ****P < 0.0001.

Immunofluorescence testing was used to determine the localization of the FAM3D protein in CRC cells (Figure 2A). Then, we knocked out the *FAM3D* gene in LoVo and HT29 cells. Sanger sequencing and Western blotting confirmed the KO efficiency of the construct (Supplementary Figure 3 and Figure 2B, 2C). Cell function tests were used to explore FAM3D’s impact on CRC progression. Colony formation and CCK-8 assays showed that *FAM3D* deficiency strongly inhibited the growth of LoVo and HT29 cells *in vitro* (Figure 2D–2F). *In vitro* migration and invasion tests showed that *FAM3D* KO cells had reduced motility (Figures 2G, 2H, 3A, 3B). To better understand the function of FAM3D, we established LoVo cells that overexpress *FAM3D* and confirmed the overexpression efficiency of the construct (Figure 3C, 3D). Conversely, increased expression of FAM3D promoted the malignant phenotypes of CRC cells (Figure 3E–3H). The results demonstrated that FAM3D promotes CRC progression *in vitro*.

### FAM3D promotes the growth of CRC cells *in vivo*

**Figure 4:**
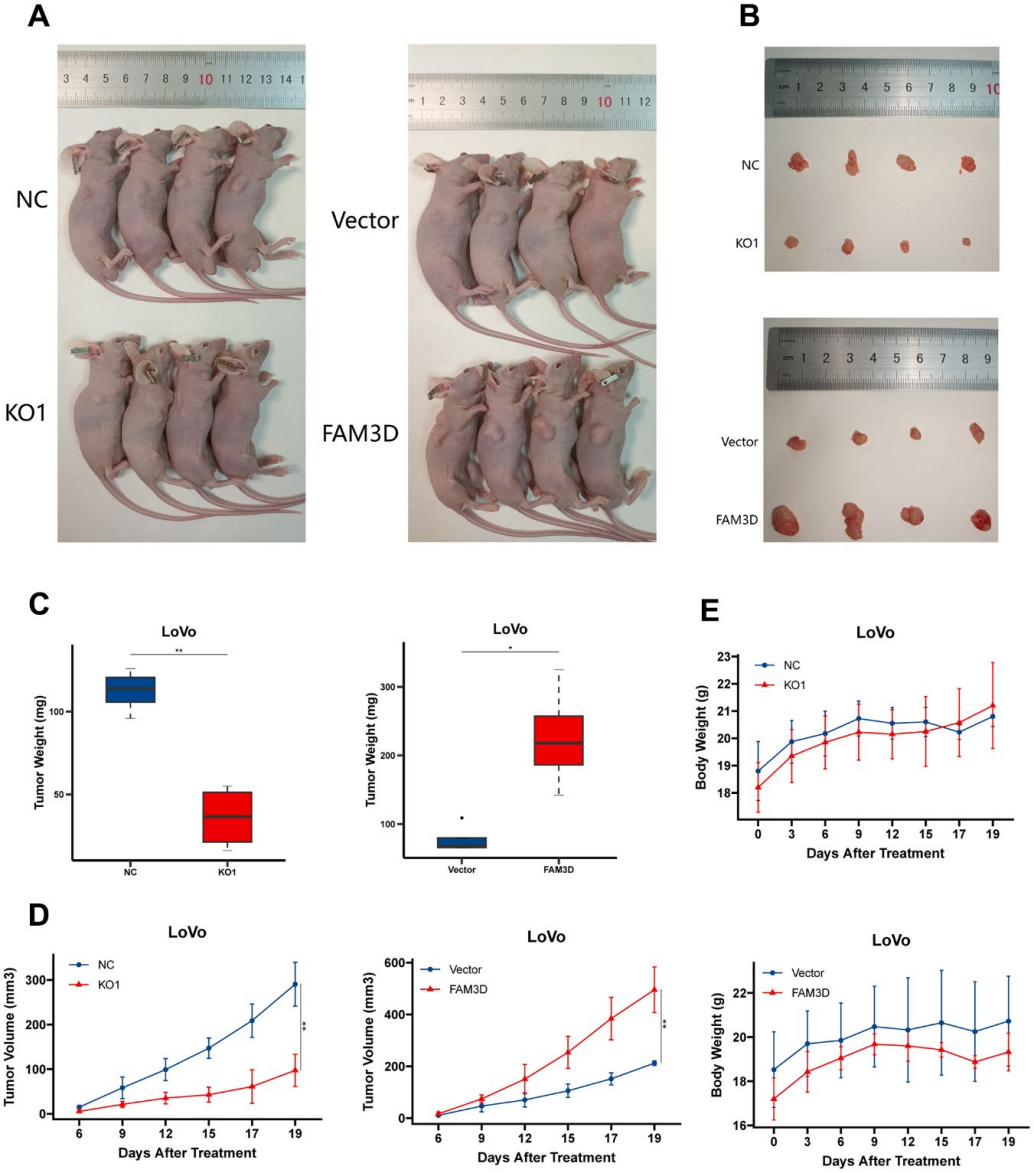
**FAM3D promotes the growth of CRC cells *in vivo*.** (**A**) Photographs of nude mice after the administration of *FAM3D* KO or *FAM3D*-overexpressing LoVo cells for 19 days. (**B**) Excised tumors on Day 19. (**C**) The weights of excised tumors on Day 19. (**D**) The tumor volumes were calculated as length × width^2^ × 0.5 every 2–3 days. (**E**) The body weights of the nude mice were recorded every 2–3 days. *P < 0.05; **P < 0.01.

To assess FAM3D’s effect on cell proliferation *in vivo*, we generated LoVo xenograft nude mouse models (Figure 4A). The results found that *FAM3D* deletion resulted in a reduction in tumor size and weight, while high expression of *FAM3D* led to an increase in both parameters (Figure 4B–4D). Moreover, the naked mice’s body weights did not vary much (Figure 4E). The data demonstrated that FAM3D enhances CRC tumor growth *in vivo*.

### *FAM3D* expression is inversely associated with *SESN2* expression

**Figure 5:**
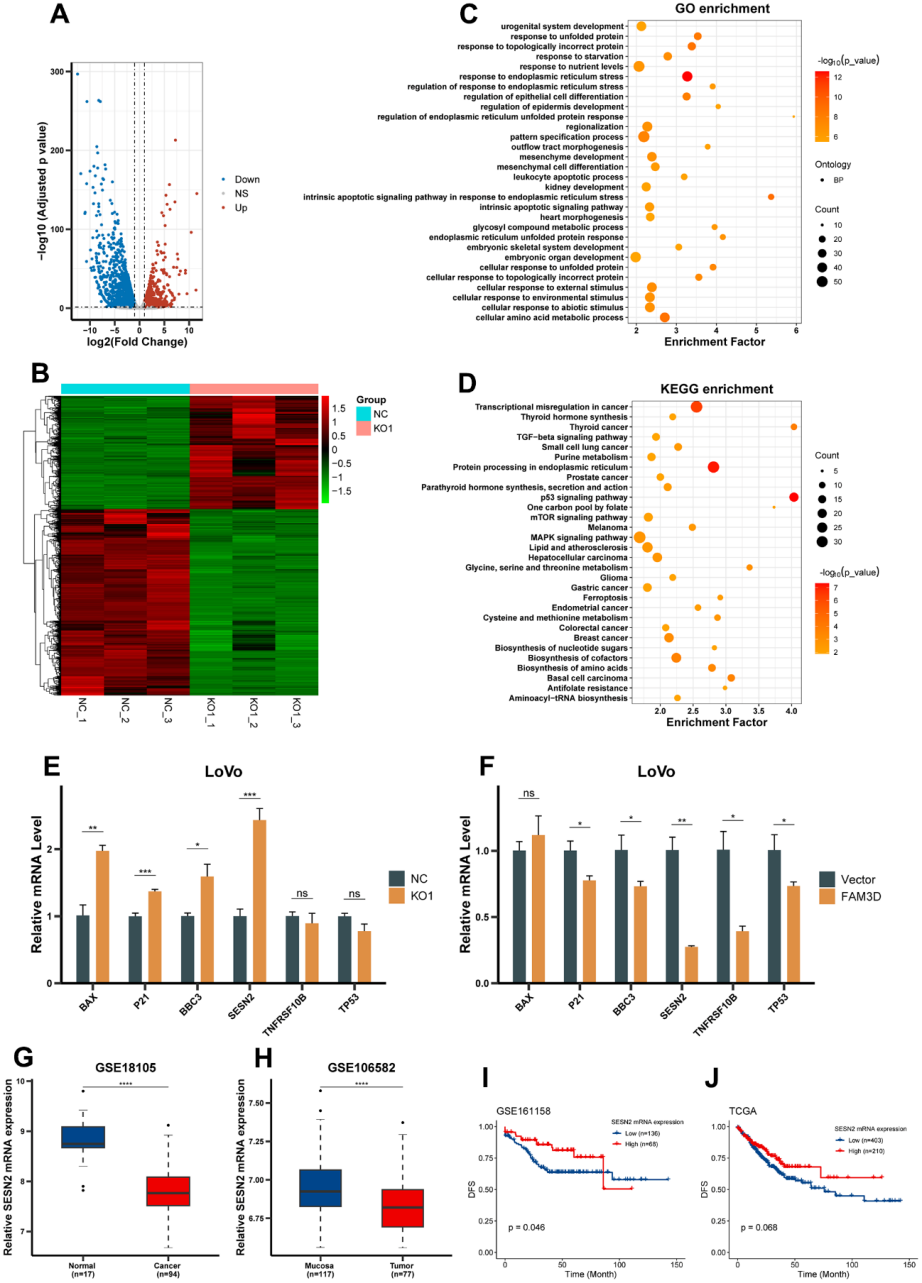
***FAM3D* expression is inversely associated with *SESN2* expression.** (**A**) Volcano plot and (**B**) hierarchical clustering heatmap showing DEGs between *FAM3D* KO and NC LoVo cells. (**C**) GO functional analysis showed the top 30 biological processes of significantly upregulated genes after *FAM3D* KO. (**D**) KEGG enrichment analysis of genes significantly upregulated after *FAM3D* KO. (**E**, **F**) RT-qPCR was used to detect changes in the expression of representative genes in the p53 and mTOR pathways in *FAM3D* (**E**) KO or (**F**) overexpressing LoVo cells. (**G**, **H**) The mRNA expression of *SESN2* in normal tissues and CRC tissues in (**G**) GSE18105 and (**H**) GSE106582. (**I**, **J**) Kaplan-Meier curves of DFS in (**I**) GSE161158 and (**J**) TCGA. *P < 0.05; **P < 0.01, ***P < 0.001, ****P < 0.0001.

Differentially expressed genes (DEGs) between *FAM3D*-knockout cells and negative control (NC) cells were detected using RNA sequencing, which found that there were 1167 upregulated genes and 1919 downregulated genes after *FAM3D* KO (Figure 5A, 5B). A focus was placed on the upregulated genes for subsequent analyses. GO analysis revealed the roles of these genes in the cell response, apoptosis, differentiation, and protein folding (Figure 5C), and KEGG analysis demonstrated these genes’ participation in pathways relevant to tumors, such as the p53 and mTOR signaling pathways (Figure 5D). Parallel analyses of CRC cells with high and low *FAM3D* expression yielded consistent results (Supplementary Figure 4A, 4B). Next, we selected six key genes associated with the p53 and mTOR pathways and further verified their expression levels, which showed that *SESN2* expression increased most significantly in *FAM3D* KO cells and decreased most significantly in *FAM3D* overexpressing cells (Figure 5E, 5F). Further analysis found that *SESN2* mRNA was downregulated in tissues of CRC (Figure 5G, 5H). Although high *SESN2* expression was associated with good CRC prognosis in GSE161158 (*P* = 0.046) (Figure 5I), the association between *SESN2* expression and CRC prognosis was only marginally significant in TCGA (*P* = 0.068) (Figure 5J). Taken together, these findings suggested that FAM3D may regulate *SESN2*, thereby promoting CRC progression.

### FAM3D functions in CRC cells by targeting *SESN2* and activating the mTORC1 pathway

**Figure 6:**
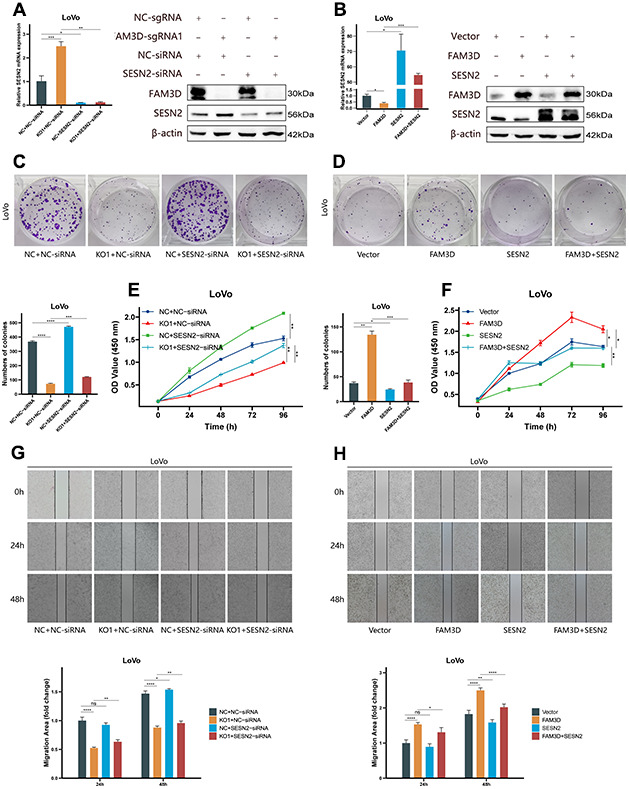
**FAM3D functions in CRC cells by targeting *SESN2* and activating the mTORC1 pathway.** (**A**) The mRNA and protein levels of *SESN2* in *FAM3D*-knockout LoVo cells with *SESN2* knockdown. (**B**) The mRNA and protein levels of *SESN2* in *FAM3D*-overexpressing LoVo cells with *SESN2* overexpression. (**C**) Colony formation assay of *FAM3D*-knockout LoVo cells with *SESN2* knockdown. (**D**) Colony formation assay of *FAM3D*-overexpressing LoVo cells with *SESN2* overexpression. (**E**) CCK-8 assay of *FAM3D*-knockout LoVo cells with *SESN2* knockdown. (**F**) CCK-8 assay of *FAM3D*-overexpressing LoVo cells with *SESN2* overexpression. (**G**) A wound healing assay of *FAM3D*-knockout LoVo cells with *SESN2* knockdown. (**H**) A wound healing assay of *FAM3D*-overexpressing LoVo cells with *SESN2* overexpression. *P < 0.05; **P < 0.01, ***P < 0.001, ****P < 0.0001.

**Figure 7:**
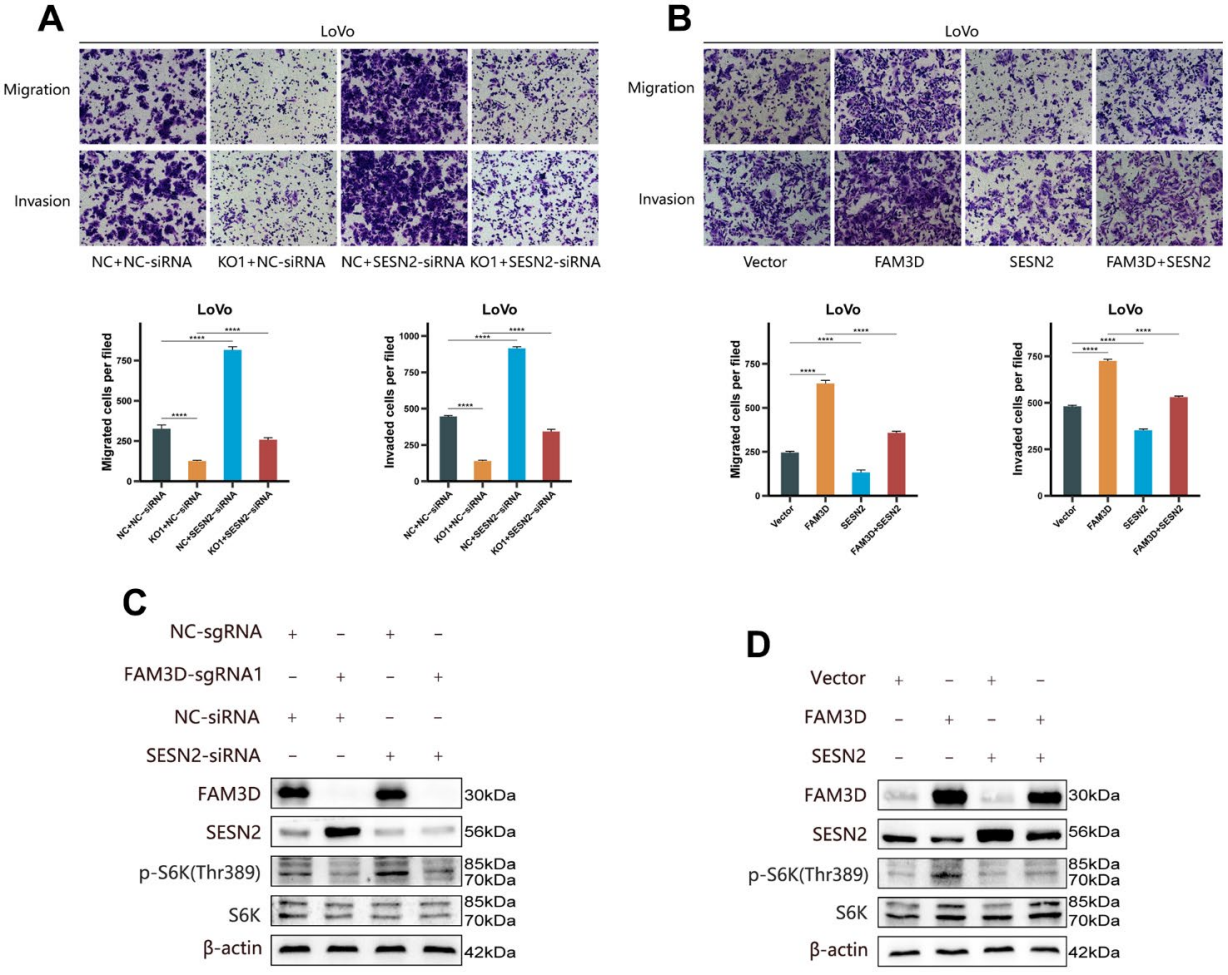
**FAM3D functions in CRC cells by targeting *SESN2* and activating the mTORC1 pathway.** (**A**) The migration and invasion of *FAM3D*-knockout LoVo cells with *SESN2* knockdown. (**B**) The migration and invasion of *FAM3D*-overexpressing LoVo cells with *SESN2* overexpression. (**C**) Western blot analysis of *FAM3D*-knockout LoVo cells with *SESN2* knockdown. (**D**) Western blot analysis of *FAM3D*-overexpressing LoVo cells with *SESN2* overexpression. ****P < 0.0001.

To verify the regulatory relationship between FAM3D and SESN2, rescue tests were performed through knocking down *SESN2* in *FAM3D* KO cells and overexpressing *SESN2* in *FAM3D* overexpression cells. Figure 6A, 6B showed that *SESN2* was successfully downregulated or upregulated, respectively. Functional experiments revealed that reducing *SESN2* expression strongly enhanced, while increasing *SESN2* expression strongly suppressed LoVo cell growth and motility; reduction of *SESN2* reversed the tumor suppressive effects of *FAM3D* KO, while upregulation of *SESN2* counteracted the oncogenic effects of high *FAM3D* expression (Figures 6C–6H, 7A, 7B). Numerous studies have revealed that SESN2 inhibits the mTORC1 pathway to have a tumor suppressor impact on many cancer types, including CRC [28, 29], endometrial cancer [30], non-small cell lung cancer [31], and neuroblastoma [32]. Thus, we performed Western blotting to explore whether FAM3D influences the mTORC1 pathway by modulating *SESN2*, which found that *FAM3D* KO reduced the phosphorylation of p70 S6K, a key marker of mTORC1 activation. This decrease was reversed by *SESN2* knockdown. Conversely, *FAM3D* overexpression increased the phosphorylation of p70 S6K, which was suppressed by *SESN2* overexpression (Figure 7C, 7D). These findings suggested that FAM3D promotes CRC progression by downregulating *SESN2* and activating the mTORC1 pathway.

### FAM3D activates the mTORC1 pathway via ATF4-mediated downregulation of *SESN2*

**Figure 8:**
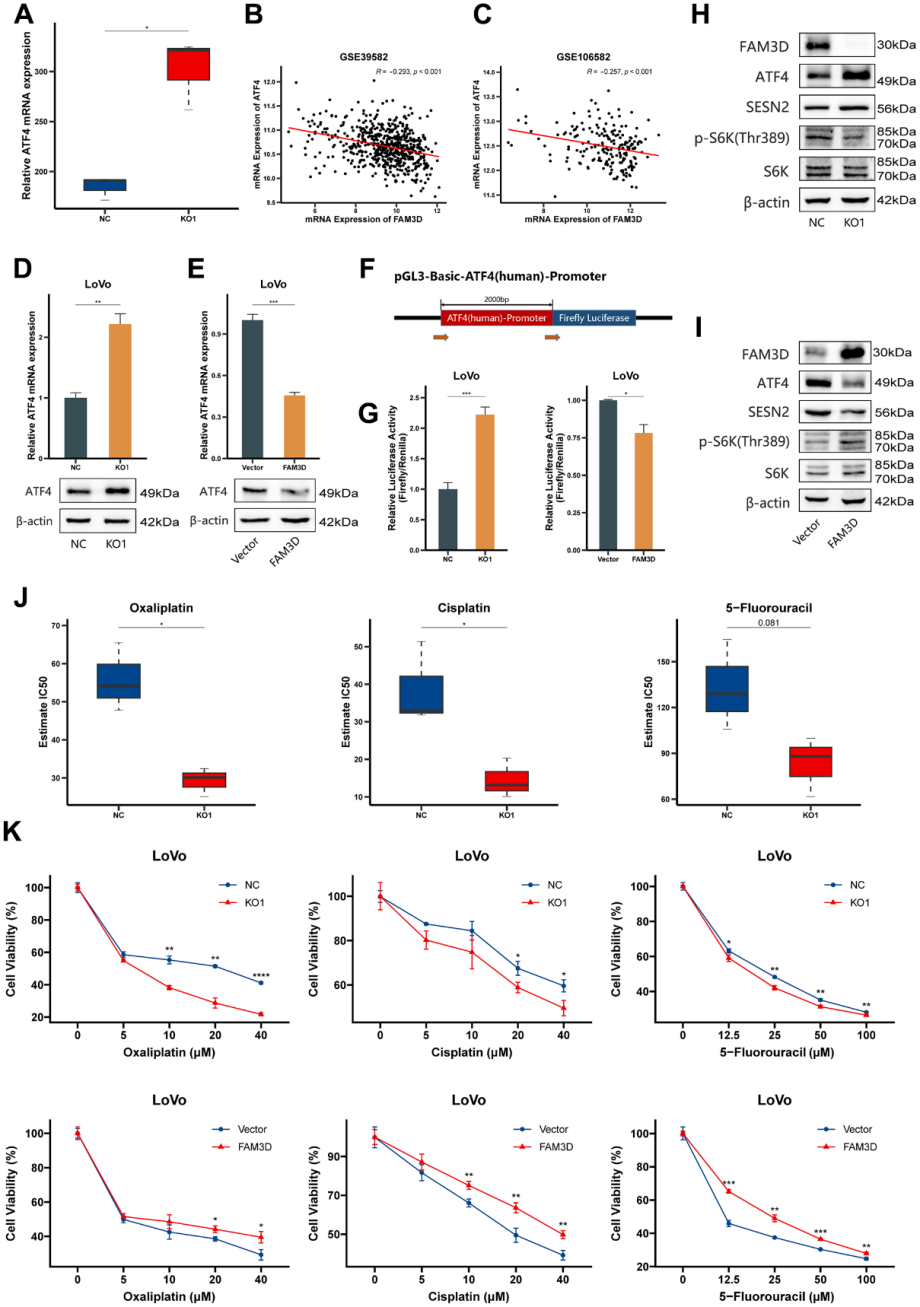
**FAM3D activates the mTORC1 pathway via ATF4-mediated downregulation of *SESN2* and reduces the chemosensitivity of CRC cells.** (**A**) The mRNA expression of *ATF4* in *FAM3D* KO and NC cells. (**B**, **C**) Correlations between *ATF4* mRNA expression and *FAM3D* mRNA expression in (**B**) GSE39582 and (**C**) GSE106582. (**D**, **E**) The mRNA and protein levels of *ATF4* in LoVo cells after *FAM3D* (**D**) KO or (**E**) overexpression. (**F**) Schematic description of the luciferase reporter. (**G**) Relative luciferase activity of the *ATF4* wild type (WT) promoter in LoVo cells with knockout or overexpression of *FAM3D*. (**H**, **I**) Western blot analysis of LoVo cells with (**H**) knockout or (**I**) overexpression of *FAM3D*. (**J**) Estimated IC_50_s of oxaliplatin, cisplatin and 5-fluorouracil in *FAM3D*-knockout and NC cells. (**K**) CCK-8 assay was used to detect the viability of *FAM3D*-knockout or *FAM3D*-overexpressing LoVo cells after treatment with a series of concentrations of oxaliplatin, cisplatin, and 5-fluorouracil. *P < 0.05; **P < 0.01, ***P < 0.001, ****P < 0.0001.

Next, we explored how FAM3D suppresses the expression of *SESN2*. Previous studies have reported that both p53 and activating transcription factor 4 (ATF4) are transcription factors of *SESN2* and can lead to its transcriptional activation in CRC [28, 33]. Thus, we analyzed *ATF4* expression in our RNA-seq and GEO data. The results showed that *ATF4* expression was significantly upregulated in *FAM3D* KO cells (Figure 8A) and was negatively correlated with *FAM3D* expression (rGSE39582 = -0.293 and rGSE106582 = -0.257) (Figure 8B, 8C), which was further confirmed by detecting *ATF4* mRNA and protein (Figure 8D, 8E). Thus, we hypothesized that FAM3D reduces *ATF4* expression by inhibiting its transcription. We performed a dual-luciferase reporter test. It was found that FAM3D caused a decrease in luciferase reporter activity regulated by the *ATF4* promoter (Figure 8F, 8G), supporting our initial hypothesis. Further, Western blotting found that *FAM3D* KO significantly upregulated ATF4 protein level, consequently increasing *SESN2* transcription and inhibiting mTORC1 (Figure 8H), while *FAM3D* overexpression played the opposite role (Figure 8I). These findings suggested that FAM3D promotes CRC progression through the ATF4-SESN2-mTORC1 pathway.

### FAM3D reduces the chemosensitivity of CRC cells

Finally, we tested whether FAM3D has an impact on the chemosensitivity of CRC cells. Using our RNA-seq results, we calculated the predicted IC_50_ concentrations of drugs or chemicals using the “oncoPredict” package (Supplementary Table 5). The results showed that *FAM3D* KO cells had lower IC_50_ values for oxaliplatin, cisplatin, and 5-fluorouracil (Figure 8J). Consistently, the CCK-8 assay revealed that *FAM3D* KO increased, whereas *FAM3D* overexpression decreased CRC cell sensitivity to three chemotherapeutic drugs (Figure 8K).

## DISCUSSION

Both aberrant hypermethylation and hypomethylation accelerate CRC progression, which is important epigenetic events that drive tumorigenesis. We have identified a 3-CpG prognostic signature consisting of three methylation-driven genes (*FAM3D*, *DAPP1*, and *PIGR*) that is an independent prognostic biomarker [13]. Here, we further demonstrated that CRC tissues and cells had a markedly downregulated expression of *FAM3D*, and that hypermethylation and low expression of *FAM3D* are independent risk factors for CRC prognosis. Mechanistically, FAM3D promoted CRC proliferation and motility through the ATF4-SESN2-mTORC1 pathway. Our results indicated that FAM3D contributes to predicting the prognosis of CRC and plays a role as an oncogene in CRC progression.

By analyzing TCGA and GEO databases, we discovered a strong negative correlation between *FAM3D* promoter methylation and its expression. Furthermore, FAM3D expression was considerably upregulated following the demethylation of CRC cells. This indicated that *FAM3D* promoter hypermethylation drives its expression silencing. The prognosis is poorer for individuals with *FAM3D* hypermethylation (cg02194211 and cg16960675) in head and neck squamous cell cancer (HNSCC) [34]. Our findings consistently showed a strong correlation between *FAM3D* hypermethylation and decreased OS and DFS in CRC patients.

Liao et al. reported that *FAM3D* expression is dramatically downregulated in cancers including gastric cancer and HNSCC [34]. Similarly, compared with adjacent cancer tissues, the expression of *FAM3D* was dramatically downregulated in CRC tissues, and FAM3D protein gradually decreased with increasing tumor stage. In addition, in many tumor types, increased FAM3D expression is linked to a favorable prognosis [34, 35]. Consistent with this conclusion, our study found that elevated *FAM3D* expression was an independent protective factor for prognosis in CRC. However, FAM3D is upregulated in some cancers and is linked to a bad prognosis. For example, FAM3D expression increases in endometrial cancer tissues compared to normal tissues [34]. As another example, patients with low-grade glioma, melanoma, and diffuse large B-cell lymphoma with high *FAM3D* expression have worse prognosis [35]. It can be seen that the expression status and prognostic significance of FAM3D in different tumors are different. The specific functions and related pathways of FAM3D in CRC are currently unclear.

Cell function experiments demonstrated that FAM3D promoted CRC cell proliferation and motility *in vitro* as well as carcinogenesis *in vivo*. This result seems to be in sharp contrast to the results of the data analysis. According to the research by Liang et al., FAM3D slows the progression of colon inflammation-related carcinogenesis in mice [25]. This seemingly contradictory phenomenon suggested that the role of FAM3D in CRC progression maybe be complex and context-dependent. One possible explanation was that FAM3D may exhibit different functions in the tumor microenvironment and the cell environment. For example, FAM3D may exert an anti-tumor effect in the CRC microenvironment, whereas in CRC cells, it promotes proliferation and motility due to the influence of other signaling pathways or factors. Additionally, because CRC is a highly heterogeneous cancer [36], different CRC cell lines and different tumor microenvironments may respond differently to FAM3D. Both hypotheses require further experimental studies to explore and verify.

Transcriptome sequencing found that, the genes whose expression was upregulated due to *FAM3D* KO were significantly enriched in the p53 and mTOR signaling pathways. Among them, the change in *SESN2* expression was the most significant. Several studies have revealed that a variety of cancers have downregulated SESN2 expression, including bladder cancer and hepatocellular carcinoma [37, 38]. Our results supported this conclusion, that is, there was less *SESN2* expression in CRC tissues compared to normal tissues. Chen et al. found that high SESN2 expression in hepatocellular carcinoma indicates a favorable prognosis (*p* = 0.003) [38]. Wei et al. analyzed the prognosis of 237 CRC patients, which found that better OS and DFS are predicted by high SESN2 expression [39]. However, our study showed that *SESN2* expression was marginally significantly correlated with CRC prognosis.

The upregulation of *SESN2* contributes to reducing ROS accumulation and inhibiting the activity of the mTORC1 pathway [40–43]. SESN2 functions as a tumor suppressor in many kinds of malignancies [44–46]. For example, downregulation of SESN2 promotes colon tumorigenesis through activating mTORC1 [29]. In addition, *SESN2* knockdown also enhances the proliferation and migration of endometrial cancer cells [30]. LSD1 inhibits mTORC1 activity by upregulating SESN2, ultimately enhancing autophagy in neuroblastoma cells [32]. We observed that *SESN2* expression was elevated in *FAM3D* KO cells but decreased in *FAM3D* overexpression cells. Moreover, *SESN2* knockdown weakened the tumor suppressor effect of *FAM3D* KO, but *SESN2* overexpression reduced the cancer-promoting effect of *FAM3D* overexpression. The possible mechanism is that FAM3D activates mTORC1 by inhibiting *SESN2* and thereby promotes CRC malignant progression. As one of the most characterized downstream effectors of mTORC1, p70 S6K phosphorylation is commonly used as a marker of mTORC1 activity [47]. Consistent with speculation, phosphorylation of p70 S6K was reduced after *FAM3D* KO and was elevated after *FAM3D* overexpression.

**Figure 9:**
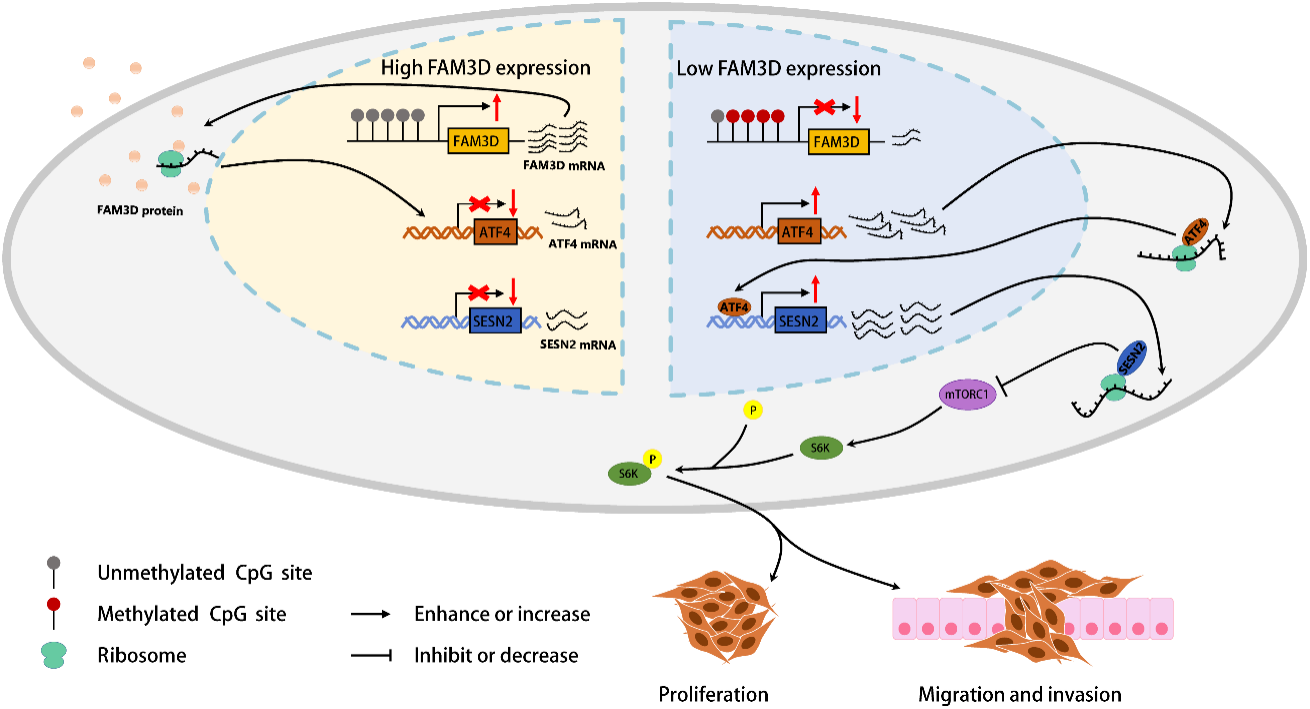
A graphical abstract of the present study.

Multiple studies have pointed out that *SESN2* is mainly regulated by p53 and ATF4 in CRC [28, 29, 33, 48, 49], and this regulation forms a negative feedback mechanism aimed at inhibiting mTORC1 activation [50]. Indeed, we discovered that *ATF4* expression were enhanced by *FAM3D* KO, but it was inhibited by *FAM3D* overexpression. And *ATF4* expression was significantly negatively correlated with *FAM3D* expression. From this we speculated that FAM3D may inhibit *SESN2* expression by inhibiting the transcription of *ATF4*. Our results fully supported the hypothesis that FAM3D inhibited the luciferase activity mediated by the *ATF4* promoter, and it promoted p70 S6K phosphorylation by reducing ATF4 and SESN2. Our findings suggested that FAM3D promotes the malignant progression of CRC through the ATF4-SESN2-mTORC1 pathway (Figure 9).

In CRC, chemotherapy failure is a major cause of disease recurrence and reduced survival. Therefore, to overcome chemotherapy resistance, new approaches to CRC treatment are required. There exists a robust correlation between FAM3D expression and tumor cell sensitivity to a variety of drugs such as elismore and lincitinib [35]. Furthermore, many studies have demonstrated that a number of drugs and chemicals exert anti-tumor effects by upregulating SESN2, including but not limited to 5-fluorouracil [33], oxaliplatin [51], nelfinavir, and bortezomib [52]. These studies could partially explain our observation that CRC cells’ sensitivity to 5-fluorouracil, oxaliplatin, and cisplatin was decreased by FAM3D. Therefore, FAM3D may serve as a new target for personalized treatment of CRC patients.

## CONCLUSIONS

*FAM3D* is lowly expressed in CRC tissues and cells, whose dysregulation may be partially linked to hypermethylation of *FAM3D* promoter. Hypermethylation and low expression of *FAM3D* are independent prognostic factors in CRC patients. Mechanistically, FAM3D activates the mTORC1 pathway through the ATF4-SESN2 axis and promotes the malignant progression of CRC (Figure 9). In addition, FAM3D reduces CRC cell sensitivity to oxaliplatin, cisplatin, and 5-fluorouracil. Together, our results indicated that FAM3D has the potential to emerge as a new target for prognosis and therapy of CRC.

## MATERIALS AND METHODS

### Data acquisition

For the TCGA database, we obtained publicly available CRC data on DNA methylation, RNA expression, and clinical information from the UCSC (https://xena.ucsc.edu/). For the CPTAC database, we downloaded COAD protein data from cBioPortal (http://cbioportal.org/). In addition, we downloaded DNA methylation (GSE131013 and GSE101764) and RNA expression (GSE18105, GSE106582, GSE17536, GSE17537, GSE39582, GSE161158, GSE44076, and GSE132465) data of CRC patients from the GEO database (https://www.ncbi.nlm.nih.gov/geo/).

### Cell culture

LoVo and LS513 cells were bought from the Cell Bank of Chinese Academy of Sciences. HT29, CW2, and HEK293T cells were acquired from Pricella Life Science & Technology Co., Ltd. LoVo, LS513, HT29, CW2, and HEK293T cells (used for generating lentivirus) were grown in Ham’s F-12K, RPMI-1640, McCoy’s 5A, DMEM, and DMEM containing 10% FBS (Procell). We cultured all cells in an incubator at 37° C and 5% CO_2_.

### RT-qPCR

After extraction with a Total RNA Kit I (Omega, R6834), Total RNA was quantified and cDNA was synthesized by ReverTra Ace® kit (Toyobo, FSQ201). The mRNA expression level was measured using SYBR green mix (Toyobo, QKD-201). Transcript levels were normalized and determined utilizing *GAPDH* and 2^-ΔΔCt^ methodology, correspondingly. The RT-qPCR primers used are listed in Supplementary Table 6.

### CRISPR/Cas9-mediated gene knockout and lentivirus-mediated overexpression

To generate *FAM3D* gene KO CRC cells, a lentivirus-based CRISPR/Cas9 system was used. We used the CRISPick (https://portals.broadinstitute.org/gppx/crispick/public) to design two single guide RNA (sgRNA) sequences targeting the human *FAM3D* gene. Then, we synthesized double-stranded sgRNA through annealing, which was further cloned into the LentiCRISPR V2 vector (Miaolingbio, plasmid no. 52961), a third-generation lentiviral backbone that coexpresses CRISPR-associated protein 9 (Cas9) and sgRNA. The recombinant LentiCRISPR V2 vector, pMD2.G (Miaolingbio, plasmid no. 12259), and pSPAX2 (Miaolingbio, plasmid no. 12260) were co-transfected into HEK239T cells with Lipofectamine™ 2000 (Invitrogen) using the recommended protocol. After continuing to culture for 48 hours, the supernatant was filtered to collect lentivirus. Subsequently, LoVo and HT29 cells were infected with the collected lentivirus. Seventy-two hours post-infection, cells were cultured in complete medium containing puromycin (LoVo: 4 μg/ml; HT29: 2 μg/ml) for selection of stable *FAM3D* KO cells. About 14 days later, to generate monoclonal cells, CRC cells with stable knockout of *FAM3D* were plated into a 96-well plate at a concentration of one cell per well. Approximately 4 weeks later, single cell–derived clones were digested and transferred to a 24-well plate for further expansion to establish pooled populations of stable *FAM3D* KO cells. Finally, the efficiency of *FAM3D* KO in each clone was assessed using Sanger sequencing and Western blotting.

In addition, we established *FAM3D*-overexpressing LoVo cells via a lentiviral expression system. *FAM3D* mRNA and protein levels were detected to verify *FAM3D* overexpression efficiency.

### Gene silencing

The negative control (sc-37007) and SESN2 (sc-106544) siRNAs were obtained from Santa Cruz Biotechnology. Lipofectamine™ 2000 was used to transfect siRNA at 25nM into LoVo cells. After incubating for an additional 48 hours, the knockdown efficiency of *SESN2* was evaluated by detecting *SESN2* mRNA and protein levels.

### Western blotting

To extract total protein, cell lysis was performed using RIPA buffer (Beyotime, P0013B) containing phosphatase inhibitor (Roche, 4906837001) and 1% PMSF (Beyotime, ST506). After quantification, 30 μg of denatured total protein was transferred to the SDS-PAGE loading well and electrophoresed at 120V for about 70 minutes. Then transfer the proteins to the PVDF membrane at a current of 200mA for about 60 minutes. After blocking, the membrane was incubated successively with primary and secondary antibodies. Finally, the proteins were developed with ECL reagent and visualized with a Tanon 5200. Differences in protein expression levels were assessed using β-actin expression as a reference. The antibodies used for Western blotting analysis included FAM3D (Proteintech, 12336-1-AP, 1:6000), SESN2 (Proteintech, 10795-1-AP, 1:6000), total S6K (Proteintech, 14485-1-AP, 1:4000), phospho-S6K-Thr389 (Proteintech, 28735-1-AP, 1:6000), ATF4 (Proteintech, 10835-1-AP, 1:1000), and β-actin (OriGene, TA811000, 1:2000).

### Detection of FAM3D protein in the cell culture supernatant

1 million cells were plated in Petri dishes. After culturing overnight, serum was withdrawn and starvation culture continued for 36 hours. The supernatant was collected and centrifuged to eliminate cells and fragments, after which the proteins were precipitated using 10% trichloroacetic acid (TCA) for 24 hours on ice. After centrifugation, the protein precipitates were washed for 3×5 minutes with acetone and dissolved in 80 μl 1×SDS-PAGE protein loading buffer. Protein levels were detected using Western blotting [53].

### Cell viability assays

Cell growth and response to chemotherapy were evaluated using CCK-8 (Dojindo). To test proliferation, LoVo and HT29 cells in logarithmic growth phase were digested and counted, then diluted to 1.5 × 10^5^ cells per ml of complete medium. Mix thoroughly and inoculate into a 96-well plate (100 μl/well), place in an incubator, and continue culturing for 4 hours. Remove the 96-well plate from the incubator and detect cell proliferation, and then detect it every 24 hours for a total of 5 times; for the chemosensitivity test, after 24 hours of pre-incubation, LoVo cells were treated with different doses of cisplatin, oxaliplatin, and 5-fluorouracil for 48 h, after which cell survival was assessed. At specified intervals, after incubation with 10% CCK-8 reagent (LoVo: 2 hours; HT29: 1 hour), a microplate reader was used to detect the optical density of the reaction solution.

### Transwell migration and invasion assays

Transwell chamber (Corning, 3422) was used to detect the *in vitro* motility of CRC cells. We resuspended 1 × 10^5^ cells in basal medium and transferred them to the top chamber, with 480 μl basal medium and 120 μl FBS in the bottom chamber. Following a 72-hour incubation, fixation was performed with 600 μl of 4% paraformaldehyde for 15 minutes. After rinsing, cells were stained with 600 μl of 0.1% crystal violet for an additional 15 minutes. Next, cells that did not pass through the holes were carefully wiped away, while the migrating cells were randomly photographed by fluorescence microscopy (LoVo: 160×magnification; HT29: 100×magnification) and counted using NIH ImageJ analysis software. For cell invasion assays, the membrane was precoated with Matrigel (BD Biosciences, 356234).

### Colony formation assay

LoVo and HT29 cells were digested, quantified, and placed in a 6-well plate (2000 cells/well). After continuing to culture for 2-3 weeks, fix cells with 4% paraformaldehyde for 15 minutes, rinse twice, stain them with crystal violet for another 15 minutes, and finally rinse them with PBS 5-6 times. The plates were allowed to dry overnight. Clones that had at least 50 cells were counted.

### Wound healing assay

Colorectal cancer cells were digested and counted, then inoculated into the 6-well plate at the same concentration and placed in an incubator to continue culturing. When the cells are all fused, use a 200-μl pipette tip to quickly make scratches. After rinsing three times, the serum was removed and starvation culture was performed. At 0, 24, and 48 hours after injury, images of the scratches were collected using fluorescence microscopy (40×magnification).

### RNA-seq and data analysis

RNA was isolated from *FAM3D* KO and NC LoVo cells. Following an assessment of the RNA quality and concentration, cDNA libraries were synthesized and sequenced on an Illumina NovaSeq 6000 by Shanghai Bioprofile Technology Company Ltd. HISAT2 was used to map the clean data to Homo sapiens GRCh38, and transcript expression was normalized by the FPKM method. DEGs were defined as follows: 1, |log_2_ fold change| ≥ 1; 2, corrected *P*-value < 0.05. The DEGs were then functionally annotated using GO, and pathway enrichment analysis was performed using KEGG.

### *In vivo* tumorigenesis assay

Beijing Vital River Laboratory Animal Technology Co., Ltd. supplied sixteen BALB/c male nude mice, aged four weeks. 3×10^6^ LoVo cells were subcutaneously inserted into the center and back regions of the right axilla of naked mice for experimental xenograft research. Subsequently, we kept feeding the mice with caution and recorded the growth of the tumors and the body weight of the naked mice every 2-3 days. On the 19th day after the tumor was implanted, all of the naked mice were killed, and the tumor tissue was taken out and weighed. The formula for calculating tumor volume was 0.5 × width^2^ × length.

### Immunofluorescence

We seeded LoVo and HT29 cells on coverslips in a 6-well plate and cultured in an incubator for approximately 12 hours. And we discarded the supernatant and fixed cells with paraformaldehyde for 15 minutes. Discarding the paraformaldehyde, cell permeabilization was performed using 0.1% Triton X-100 for 10 minutes. After blocking on a shaker, we incubated cells with FAM3D primary antibody (Affinity, DF4832, 1:300) overnight at 4° C. Incubating cells with a CoraLite 594-conjugated secondary antibody (Proteintech, SA00013-4, 1:300) was performed for 60 minutes on a shaker in the dark. Then, cells on coverslips were stained using DAPI (Beyotime, C1005). Finally, we used fluorescence microscopy to capture blue and red fluorescence in the same field of view (160×magnification).

### Dual-luciferase reporter assay

*ATF4* promoter was subcloned and inserted a luciferase reporter plasmid (pGL3). After being cultivated for 12 hours, LoVo cells were co-transfected with the recombinant plasmid pGL3 and pGL4.75. After 48 hours, the experiments were carried out by a dual-luciferase reporter assay kit (Promega, E1910). Finally, we used the microplate reader to detect the signal intensity of luciferin.

### Statistical analysis

Unless otherwise stated, three independent replicates of the experiment were performed to generate data. All analyzes were carried out using R 4.2.2. Variances among groups were estimated using one-way ANOVAs, Student’s t tests, Pearson and Spearman correlation tests, Wilcoxon rank sum tests, and χ^2^ tests. Survival probabilities were determined using the Kaplan–Meier curve alongside the log-rank examination. Prognostically relevant variables were identified by Cox proportional hazards models. *P* < 0.05 was considered to be statistically different.

## Supplementary Material

Supplementary Figures

Supplementary Tables 1-4

Supplementary Table 5

Supplementary Table 6
