# Supplementary material for: DNA methylation-driven gene FAM3D promotes colorectal cancer growth via the ATF4-SESN2-mTORC1 pathway
Source: Aging (Albany NY). 2024 Oct 10;16(19):12866–92. doi: 10.18632/aging.206115 (PMC11501385; doi:10.18632/aging.206115)
Supplement: Supplementary Figures [file aging-16-206115-s001.pdf]

## SUPPLEMENTARY FIGURES

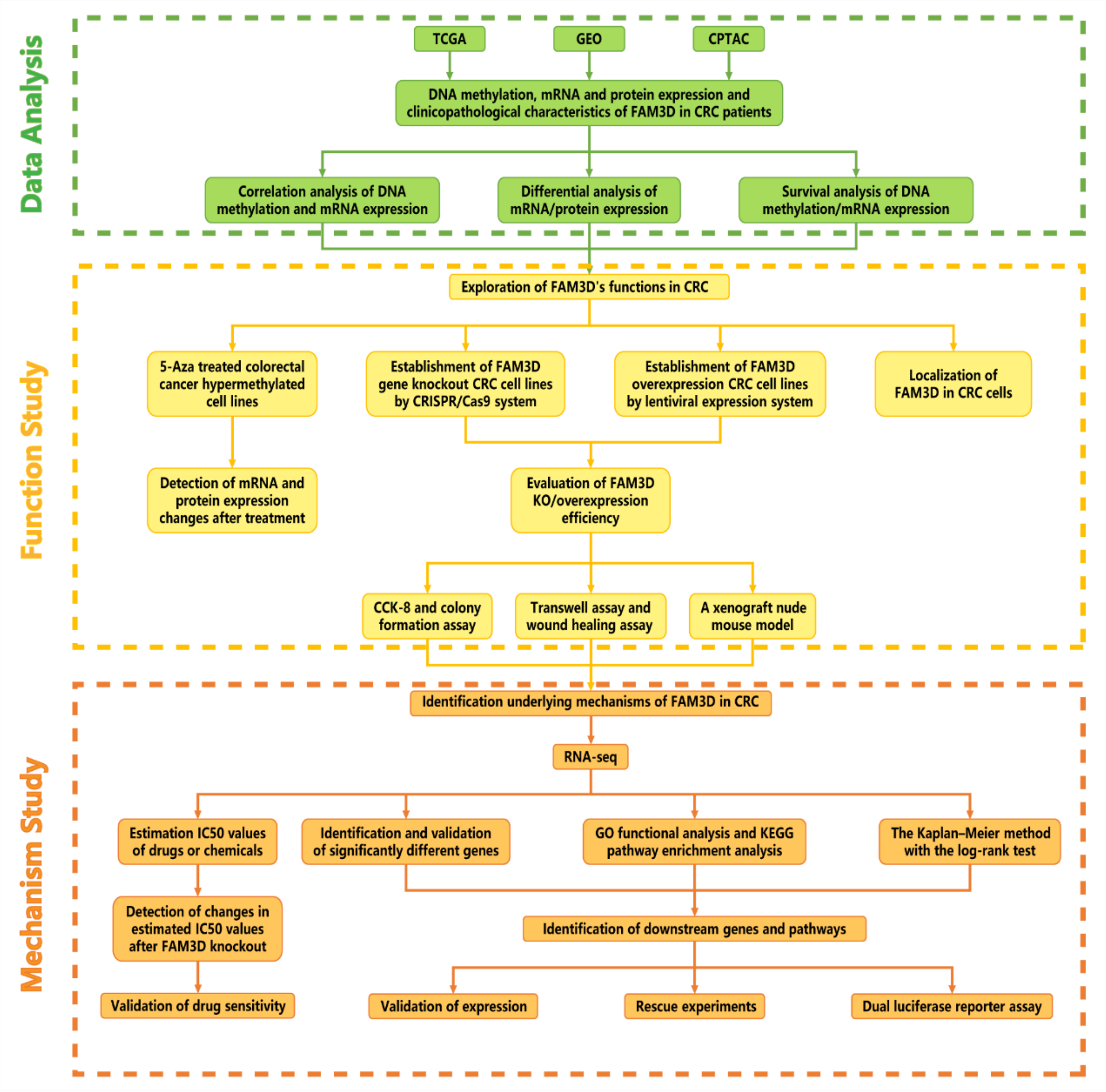


**Supplementary Figure 1. A workflow of the present study.**

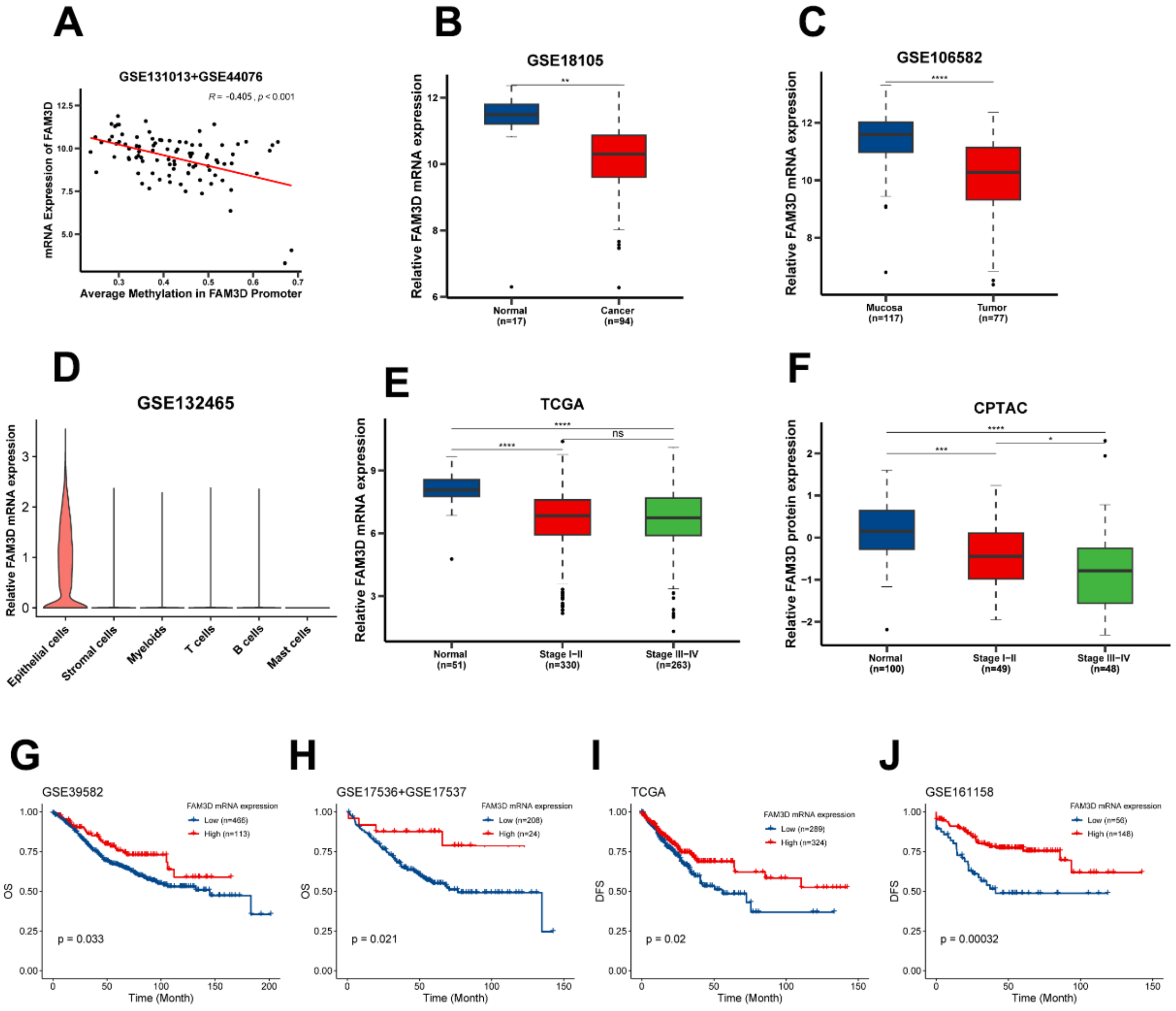


**Supplementary Figure 2. Methylation, expression and prognosis of *FAM3D*.** (**A**) Correlation between methylation of *FAM3D* promoter and its mRNA expression in GEO cohorts (GSE131013 + GSE44076). (**B**, **C**) The mRNA expression of *FAM3D* in adjacent normal tissues and CRC tissues in (**B**) GSE18105 and (**C**) GSE106582. (**D**) *FAM3D* mRNA expression in different cell types in GSE132465. (**E**, **F**) *FAM3D* mRNA and protein expression in different tumor stages in TCGA (**E**, mRNA expression) and CPTAC (**F**, protein expression). (**G**, **H**) Kaplan-Meier curve of OS based on the *FAM3D* expression in tumor tissues of GEO cohorts (**G**: GSE39582; **H**: GSE17536 + GSE17537). (**I**, **J**) Kaplan-Meier curve of DFS based on the *FAM3D* expression in tumor tissues in (**I**) TCGA and (**J**) GSE161158. *$P < 0.05$; **$P < 0.01$, ***$P < 0.001$, ****$P < 0.0001$.

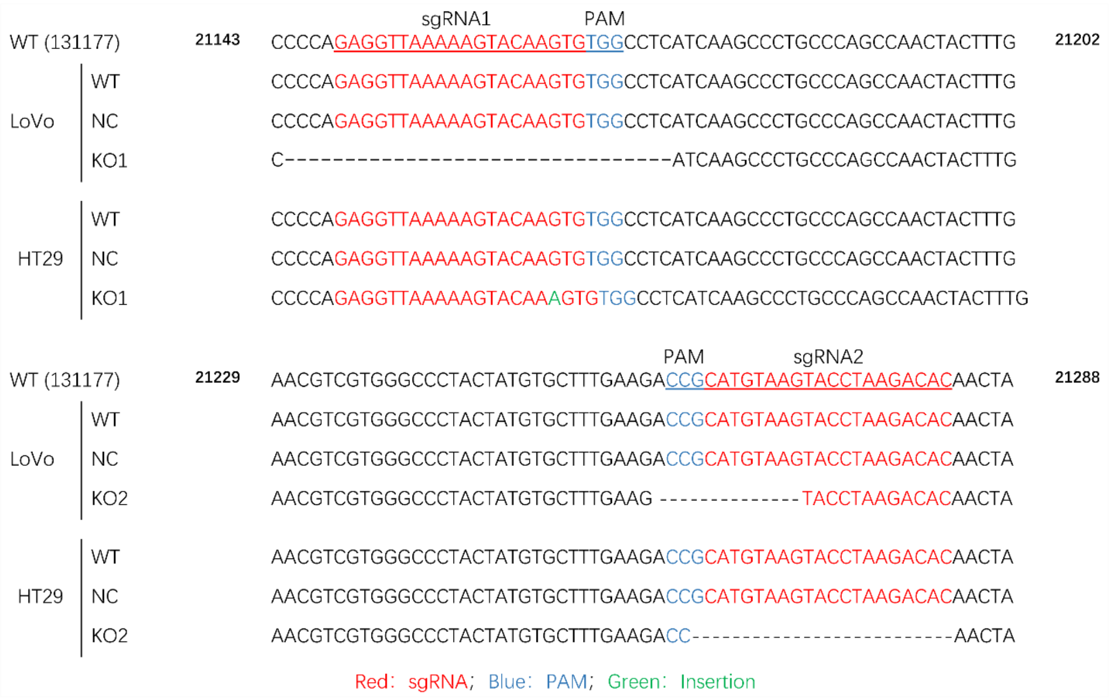


**Supplementary Figure 3. Genomic DNAs from four cell types (WT *FAM3D*, NC, KO1, and KO2 cells) of LoVo and HT29 cells were isolated and PCR amplicons flanking the CRISPR/Cas9-targeted regions were sequenced.** Sequence alignments of bases 21143–21202 and 21229–21288 of the *FAM3D* gene are shown. Numbering is based on the entire *FAM3D* gene sequence (NCBI Gene: 131177).

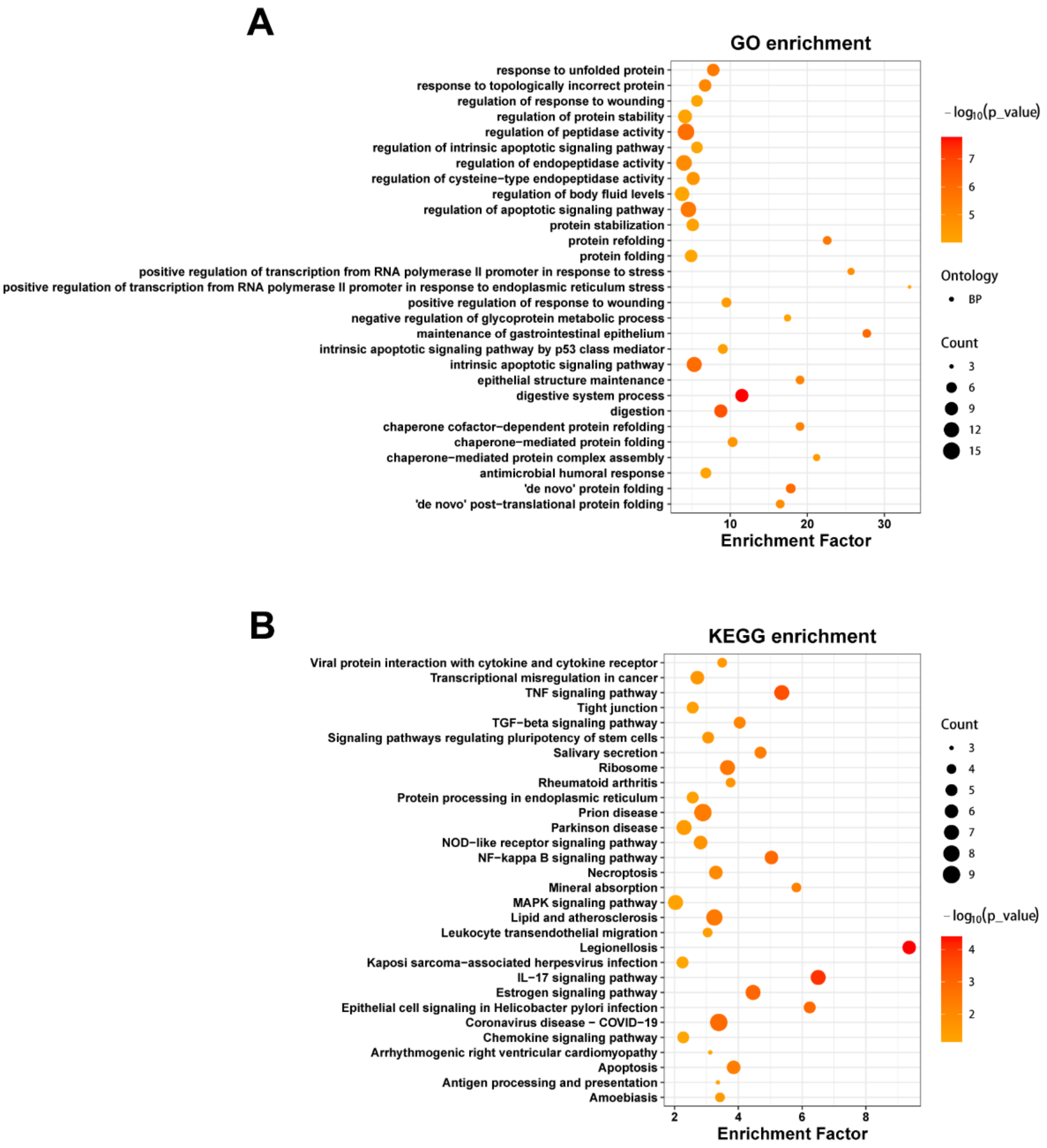


**Supplementary Figure 4. Bioinformatics analysis for *FAM3D* overexpression CRC cells in GSE132465.** (**A**) GO functional analysis showed the top 29 biological processes that are enriched in DEGs. (**B**) KEGG enrichment analysis showed the top 30 signaling pathways that are enriched in DEGs.
