# Supplementary material for: DNA methylation-driven gene FAM3D promotes colorectal cancer growth via the ATF4-SESN2-mTORC1 pathway
Source: Aging (Albany NY). 2024 Oct 10;16(19):12866–92. doi: 10.18632/aging.206115 (PMC11501385; doi:10.18632/aging.206115)
Supplement: Supplementary Tables 1-4 [file aging-16-206115-s002.pdf]

SUPPLEMENTARY TABLES

Supplementary Table 1. Associations of *FAM3D* methylation and CRC overall survival in TCGA.

Variables	Number (%)	Univariate Cox		Multivariate Cox	
		HR (95% CI)	*P*-Value	HR_{adj} (95% CI)	*P*-Value
**Age**					
Age ≥ 60 years	199 (64.61%)	2.02 (1.12-3.63)	**0.019**	2.52 (1.38-4.58)	**0.003**
Age < 60 years	109 (35.39%)				
**Gender**					
Male	166 (53.90%)	1.16 (0.72-1.87)	0.55	-	-
Female	142 (46.10%)				
**T stage**					
T3-4	257 (83.44%)	2.92 (1.06-8.05)	**0.038**	1.50 (0.52-4.31)	0.45
T1-2	51 (16.56%)				
**N stage**					
N1-3	133 (43.18%)	2.89 (1.76-4.75)	**2.88e-05**	2.14 (1.21-3.79)	**0.009**
N0	175 (56.82%)				
**M stage**					
M1	51 (16.56%)	3.50 (2.09-5.86)	**1.91e-06**	2.70 (1.50-4.86)	**0.001**
M0	257 (83.44%)				
***FAM3D* methylation**					
High	71 (23.05%)	2.22 (1.35-3.63)	**0.002**	2.02 (1.23-3.34)	**0.006**
Low	237 (76.95%)				

Supplementary Table 2. Associations of *FAM3D* mRNA and CRC overall survival in TCGA.

Variables	Number (%)	Univariate Cox		Multivariate Cox	
		HR (95% CI)	*P*-Value	HR_{adj} (95% CI)	*P*-Value
**Age**					
Age ≥ 60 years	337 (72.16%)	1.92(1.12-3.31)	**0.018**	2.75 (1.57-4.80)	**4.05e-04**
Age < 60 years	130 (27.84%)				
**Gender**					
Male	246 (52.68%)	0.9879649 (0.65-1.50)	0.955	-	-
Female	221 (47.32%)				
**T stage**					
T3-4	374 (80.09%)	3.58 (1.45-8.84)	**0.006**	2.43 (0.96-6.14)	0.061
T1-2	93 (19.91%)				
**N stage**					
N1-3	197 (42.18%)	3.16 (2.03-4.91)	**3.09e-07**	1.96 (1.15-3.35)	**0.013**
N0	270 (57.82%)				
**M stage**					
M1	72 (15.42%)	4.50 (2.91-6.94)	**1.13e-11**	3.24 (1.95-5.39)	**5.55e-06**
M0	395 (84.58%)				
***FAM3D* mRNA**					
High	297 (63.60%)	0.56 (0.37-0.84)	**0.006**	0.51 (0.33-0.78)	**0.002**
Low	170 (36.40%)				

Supplementary Table 3. Associations of *FAM3D* mRNA and CRC overall survival in GSE39582.

Variables	Number (%)	Univariate Cox		Multivariate Cox	
		HR (95% CI)	*P*-Value	HR_{adj} (95% CI)	*P*-Value
**Age**					
Age ≥ 60 years	425 (73.53%)	1.41 (0.99-1.99)	0.052	-	-
Age < 60 years	153 (26.47%)				
**Gender**					
Male	319 (55.09%)	1.31 (0.98-1.75)	0.067	-	-
Female	260 (44.91%)				
**Stage**					
III-IV	273 (47.15%)	1.80 (1.35-2.39)	**5.62e-05**	1.78 (1.34-2.36)	**8.01e-05**
I-II	306 (52.85%)				
***FAM3D* mRNA**					
High	113 (19.52%)	0.65 (0.44-0.97)	**0.033**	0.67 (0.45-0.99)	**0.049**
Low	466 (80.48%)				

Supplementary Table 4. Associations of *FAM3D* mRNA and CRC disease free survival in GSE161158.

Variables	Number (%)	Univariate Cox		Multivariate Cox	
		HR (95% CI)	*P*-Value	HR_{adj} (95% CI)	*P*-Value
**Age**					
Age ≥ 60 years	135 (66.18%)	0.60 (0.36-1.00)	0.052	-	-
Age < 60 years	69 (33.82%)				
**Stage**					
III-IV	97 (47.55%)	3.62 (2.04-6.43)	**1.15e-05**	3.51 (1.98-6.24)	**1.84e-05**
I-II	107 (52.45%)				
***FAM3D* mRNA**					
High	148 (72.55%)	0.40 (0.24-0.67)	**4.94e-04**	0.42 (0.25-0.70)	**0.001**
Low	56 (27.45%)				
