# Supplementary material for: DNA methylation-driven gene FAM3D promotes colorectal cancer growth via the ATF4-SESN2-mTORC1 pathway
Source: Aging (Albany NY). 2024 Oct 10;16(19):12866–92. doi: 10.18632/aging.206115 (PMC11501385; doi:10.18632/aging.206115)
Supplement: Supplementary Table 5 [file aging-16-206115-s003.docx]

**Supplementary Table 5. Estimated IC_50_ of each drug or chemical for NC and KO1 LoVo cells.**

| Drug | NC_1 | NC_2 | NC_3 | KO1_1 | KO1_2 | KO1_3 |
| --- | --- | --- | --- | --- | --- | --- |
| VE821 | 101.393 | 104.105 | 107.278 | 33.163 | 29.898 | 35.501 |
| Nilotinib | 54.367 | 55.511 | 51.909 | 26.569 | 23.051 | 26.275 |
| Linsitinib | 53.525 | 54.785 | 53.700 | 33.889 | 31.541 | 32.132 |
| LCL161 | 124.886 | 125.508 | 129.398 | 150.971 | 151.740 | 155.033 |
| KU.55933 | 95.955 | 93.989 | 91.512 | 69.462 | 73.257 | 74.527 |
| Dactolisib | 0.347 | 0.334 | 0.389 | 0.113 | 0.082 | 0.149 |
| Lapatinib | 31.485 | 29.953 | 32.942 | 12.661 | 11.699 | 12.433 |
| PD173074 | 103.419 | 89.642 | 101.903 | 38.628 | 30.546 | 32.550 |
| Tozasertib | 23.411 | 22.700 | 21.031 | 13.357 | 14.859 | 12.808 |
| Navitoclax | 12.202 | 13.383 | 13.749 | 3.344 | 3.826 | 3.443 |
| Rapamycin | 0.224 | 0.195 | 0.190 | 0.070 | 0.055 | 0.076 |
| Axitinib | 29.341 | 27.817 | 26.260 | 17.654 | 17.966 | 16.061 |
| EPZ5676 | 321.445 | 318.664 | 356.734 | 193.407 | 176.438 | 201.682 |
| Teniposide | 0.885 | 0.966 | 1.278 | 2.114 | 2.410 | 2.520 |
| Tamoxifen | 47.284 | 43.806 | 51.287 | 29.477 | 24.522 | 25.488 |
| BMS.754807 | 2.788 | 2.496 | 2.738 | 1.525 | 1.177 | 1.591 |
| NVP.ADW742 | 24.789 | 22.277 | 27.925 | 9.939 | 7.459 | 10.504 |
| Niraparib | 65.772 | 67.888 | 70.716 | 81.123 | 87.771 | 84.905 |
| Vorinostat | 5.317 | 4.705 | 5.190 | 2.949 | 3.640 | 2.994 |
| LJI308 | 232.246 | 240.643 | 233.173 | 99.463 | 131.698 | 110.228 |
| Nutlin.3a.... | 170.388 | 146.843 | 180.526 | 62.796 | 56.804 | 71.172 |
| Palbociclib | 77.774 | 65.226 | 88.677 | 19.037 | 12.503 | 25.403 |
| AZD4547 | 26.918 | 25.335 | 30.907 | 13.349 | 12.327 | 10.796 |
| Sorafenib | 17.263 | 17.492 | 19.902 | 11.212 | 10.075 | 10.697 |
| Cediranib | 12.622 | 10.228 | 12.923 | 6.935 | 4.780 | 6.357 |
| GSK269962A | 23.383 | 23.509 | 22.449 | 15.944 | 12.690 | 14.764 |
| Gallibiscoquinazole | 15.869 | 16.755 | 17.134 | 11.038 | 12.292 | 9.673 |
| MK.2206 | 32.301 | 31.569 | 38.514 | 13.105 | 7.693 | 18.660 |
| Luminespib | 0.116 | 0.118 | 0.138 | 0.063 | 0.069 | 0.088 |
| MIRA.1 | 290.525 | 276.629 | 289.443 | 204.072 | 171.442 | 160.892 |
| AT13148 | 63.068 | 74.157 | 59.469 | 22.015 | 22.898 | 24.828 |
| BMS.536924 | 10.500 | 9.515 | 11.130 | 6.902 | 4.963 | 6.961 |
| Sepantronium.bromide | 0.008 | 0.008 | 0.008 | 0.024 | 0.021 | 0.019 |
| GSK2578215A | 174.126 | 173.056 | 177.899 | 118.647 | 92.271 | 108.792 |
| ABT737 | 11.710 | 13.994 | 14.206 | 6.128 | 6.748 | 6.600 |
| Mitoxantrone | 1.118 | 1.141 | 1.676 | 2.232 | 2.453 | 2.894 |
| Ruxolitinib | 148.720 | 145.355 | 135.130 | 121.052 | 120.083 | 107.554 |
| Cytarabine | 7.172 | 6.559 | 8.695 | 3.423 | 4.220 | 3.019 |
| Paclitaxel | 0.090 | 0.069 | 0.075 | 0.037 | 0.043 | 0.035 |
| Crizotinib | 33.395 | 31.262 | 30.663 | 15.272 | 22.284 | 17.235 |
| Cyclophosphamide | 200.302 | 186.989 | 185.130 | 167.168 | 151.494 | 144.940 |
| Fulvestrant | 78.314 | 77.385 | 78.223 | 111.275 | 104.838 | 119.045 |
| I.BRD9 | 108.283 | 101.004 | 102.691 | 69.872 | 46.092 | 57.661 |
| GSK1904529A | 92.278 | 90.498 | 92.123 | 66.697 | 62.965 | 53.269 |
| MG.132 | 0.301 | 0.237 | 0.286 | 0.148 | 0.136 | 0.157 |
| AZD6482 | 30.983 | 34.090 | 28.230 | 21.592 | 16.057 | 22.793 |
| P22077 | 155.258 | 110.390 | 123.343 | 70.723 | 47.624 | 54.067 |
| NU7441 | 15.640 | 15.509 | 14.379 | 12.756 | 13.758 | 12.942 |
| Foretinib | 4.853 | 3.280 | 4.542 | 1.199 | 1.966 | 1.664 |
| RVX.208 | 135.486 | 127.505 | 141.917 | 104.362 | 83.033 | 105.888 |
| RO.3306 | 22.192 | 22.462 | 20.679 | 18.729 | 18.979 | 18.264 |
| Savolitinib | 18.560 | 15.602 | 15.501 | 11.742 | 10.359 | 11.894 |
| PLX.4720 | 128.080 | 129.362 | 182.782 | 55.698 | 36.988 | 65.646 |
| Pevonedistat | 1.212 | 1.161 | 1.122 | 2.916 | 3.215 | 2.363 |
| OSI.027 | 154.911 | 130.036 | 177.034 | 73.343 | 84.296 | 108.890 |
| Temozolomide | 425.770 | 409.912 | 456.097 | 362.281 | 346.868 | 360.873 |
| Wnt.C59 | 77.677 | 72.106 | 79.600 | 66.548 | 55.689 | 62.383 |
| VE.822 | 37.178 | 29.934 | 36.948 | 23.481 | 20.813 | 26.425 |
| Oxaliplatin | 54.172 | 47.770 | 65.501 | 30.119 | 25.114 | 32.402 |
| BPD.00008900 | 109.162 | 104.179 | 106.727 | 81.791 | 69.671 | 86.157 |
| Pictilisib | 6.461 | 5.300 | 6.231 | 2.881 | 1.653 | 3.955 |
| Ibrutinib | 80.349 | 70.885 | 84.805 | 122.155 | 107.320 | 99.290 |
| Buparlisib | 3.237 | 2.995 | 3.206 | 2.181 | 1.685 | 2.401 |
| Uprosertib | 28.902 | 30.251 | 30.418 | 9.835 | 4.959 | 16.672 |
| MN.64 | 98.566 | 93.206 | 90.517 | 149.651 | 121.768 | 135.670 |
| Topotecan | 0.477 | 0.453 | 0.737 | 1.675 | 2.200 | 2.791 |
| UMI.77 | 11.861 | 10.939 | 10.629 | 18.878 | 22.801 | 17.421 |
| AZD8055 | 0.937 | 0.923 | 0.930 | 0.736 | 0.704 | 0.813 |
| GSK343 | 17.564 | 17.566 | 18.277 | 16.164 | 14.237 | 15.381 |
| Ribociclib | 50.813 | 50.901 | 50.343 | 45.998 | 42.641 | 45.965 |
| Podophyllotoxin.bromide | 0.553 | 0.566 | 0.705 | 0.400 | 0.354 | 0.424 |
| MK.1775 | 1.840 | 1.872 | 2.098 | 1.109 | 1.603 | 1.198 |
| Bortezomib | 0.010 | 0.008 | 0.010 | 0.007 | 0.005 | 0.007 |
| Docetaxel | 0.014 | 0.010 | 0.014 | 0.007 | 0.007 | 0.008 |
| I.BET.762 | 39.557 | 27.973 | 35.593 | 21.499 | 17.886 | 23.116 |
| VX.11e | 26.777 | 17.059 | 25.051 | 7.630 | 14.530 | 12.434 |
| GDC0810 | 156.617 | 157.097 | 159.353 | 131.640 | 99.375 | 107.711 |
| AGI.5198 | 96.036 | 91.993 | 96.132 | 126.750 | 110.495 | 112.527 |
| PD0325901 | 1.354 | 1.051 | 1.256 | 1.869 | 1.790 | 2.449 |
| Fludarabine | 158.831 | 197.930 | 213.432 | 119.846 | 105.853 | 153.892 |
| Cisplatin | 32.914 | 31.803 | 51.379 | 10.097 | 20.329 | 13.148 |
| MK.8776 | 24.952 | 27.953 | 29.827 | 20.252 | 16.260 | 23.405 |
| GNE.317 | 2.264 | 2.165 | 2.427 | 1.306 | 0.842 | 1.696 |
| Dabrafenib | 133.437 | 106.934 | 154.675 | 76.235 | 50.874 | 99.121 |
| Acetalax | 251.027 | 167.010 | 183.172 | 104.550 | 140.334 | 58.300 |
| Staurosporine | 0.067 | 0.052 | 0.061 | 0.025 | 0.045 | 0.042 |
| Alpelisib | 43.975 | 45.280 | 49.315 | 23.544 | 19.368 | 36.182 |
| Zoledronate | 40.639 | 39.603 | 38.946 | 49.332 | 44.982 | 43.977 |
| GSK591 | 126.845 | 120.069 | 112.951 | 68.482 | 100.096 | 64.708 |
| Daporinad | 0.021 | 0.015 | 0.015 | 0.008 | 0.012 | 0.006 |
| LGK974 | 50.925 | 47.867 | 46.905 | 79.383 | 64.604 | 62.724 |
| Taselisib | 11.016 | 10.543 | 13.325 | 5.887 | 3.267 | 8.934 |
| Selumetinib | 68.793 | 65.646 | 66.366 | 62.950 | 59.534 | 64.180 |
| Ulixertinib | 20.793 | 16.821 | 16.680 | 13.236 | 14.846 | 12.449 |
| WIKI4 | 38.164 | 38.046 | 40.535 | 44.447 | 40.510 | 43.670 |
| KRAS..G12C..Inhibitor.12 | 74.236 | 68.683 | 75.547 | 98.325 | 82.933 | 85.306 |
| GSK2606414 | 53.469 | 43.321 | 53.090 | 38.122 | 27.800 | 41.963 |
| Dactinomycin | 0.089 | 0.083 | 0.100 | 0.078 | 0.064 | 0.078 |
| BDP.00009066 | 12.392 | 11.397 | 11.179 | 8.900 | 7.412 | 10.366 |
| Dactinomycin | 0.009 | 0.007 | 0.011 | 0.006 | 0.005 | 0.007 |
| Sabutoclax | 0.547 | 0.498 | 0.577 | 0.739 | 0.703 | 0.956 |
| SB216763 | 249.633 | 252.083 | 196.688 | 138.390 | 205.915 | 126.763 |
| YK.4.279 | 10.552 | 8.470 | 10.246 | 8.089 | 7.498 | 7.570 |
| Sapitinib | 38.784 | 35.627 | 41.246 | 83.893 | 51.390 | 79.532 |
| LY2109761 | 142.382 | 125.670 | 139.199 | 272.058 | 178.582 | 217.917 |
| X5.Fluorouracil | 164.551 | 105.752 | 129.118 | 88.005 | 61.722 | 99.783 |
| OTX015 | 16.720 | 10.984 | 15.278 | 9.305 | 8.226 | 9.662 |
| PFI3 | 195.004 | 204.628 | 209.670 | 187.877 | 154.706 | 179.843 |
| Camptothecin | 0.061 | 0.047 | 0.084 | 0.083 | 0.149 | 0.116 |
| Leflunomide | 147.887 | 137.832 | 136.732 | 151.765 | 149.325 | 152.008 |
