# Supplementary material for: DNA methylation-driven gene FAM3D promotes colorectal cancer growth via the ATF4-SESN2-mTORC1 pathway
Source: Aging (Albany NY). 2024 Oct 10;16(19):12866–92. doi: 10.18632/aging.206115 (PMC11501385; doi:10.18632/aging.206115)
Supplement: Supplementary Table 6 [file aging-16-206115-s004.pdf]

Supplementary Table 6. Primers used in the study.

Primer	Sequence (5' - 3')
Clone primers	
CDS_*FAM3D*_F[a]	CTAGCCTGATACGAACTCGGATGAGAG TGTCAGGTGTGCT
CDS_*FAM3D*_R[b]	CGTCATGGTCTTTGTAGTCGAAATGGC TTCGGGGGCATGC
RT-qPCR primers	
*FAM3D*_F	ATGAGAGTGTCAGGTGTGCTTC
*FAM3D*_R	CTTGATGAGGCCACACTTGTAC
*SESN2*_F	AAGGACTACCTGCGGTTCG
*SESN2*_R	CGCCCAGAGGACATCAGTG
*GAPDH*_F	GGAGCGAGATCCCTCCAAAAT
*GAPDH*_R	GGCTGTTGTCATACTTCTCATGG
*BAX*_F	CCCGAGAGGTCTTTTTCCGAG
*BAX*_R	CCAGCCCATGATGGTTCTGAT
*P21*_F	TGTCCGTCAGAACCCATGC
*P21*_R	AAAGTCGAAGTTCCATCGCTC
*BBC3*_F	GACCTCAACGCACAGTACGAG
*BBC3*_R	AGGAGTCCCATGATGAGATTGT
*TNFRSF10B*_F	ATGGAACAACGGGGACAGAAC
*TNFRSF10B*_R	CTGCTGGGGAGCTAGGTCT
*TP53*_F	CAGCACATGACGGAGGTTGT
*TP53*_R	TCATCCAAATACTCCACACGC
*ATF4*_F	CCCTTCACCTTCTTACAACCTC
*ATF4*_R	TGCCCAGCTCTAAACTAAAGGA
Gene knockout primers	
*FAM3D*_sgRNA1_F	CACCGGAGGTTAAAAAGTACAAGTG
*FAM3D*_sgRNA1_R	AAACCACTTGTACTTTTTAACCTCC
*FAM3D*_sgRNA2_F	CACCGGTGTCTTAGGTACTTACATG
*FAM3D*_sgRNA2_R	AAACCATGTAAGTACCTAAGACACC

[a]suffix "F" represents forward primer; [b]suffix "R" represents reverse primer.
